# A QUALITATIVE STUDY OF NURSING HOME STAFF CONCEPTUALIZATION OF EVERYDAY DECISION-MAKING CAPACITY

**DOI:** 10.1093/geroni/igz038.2576

**Published:** 2019-11-08

**Authors:** Whitney L Mills, Mark E Kunik, Lea Kiefer, Hannah Curren-Vo, Amy Mochel, Aanand Naik

**Affiliations:** 1 Center for Innovation in Long-Term Services and Supports, Providence VA Medical Center, Providence, Rhode Island, United States; 2 Baylor College of Medicine, Houston, Texas, United States; 3 Michael E. DeBakey VA Medical Center, Houston, Texas, United States; 4 Center for Innovations in Quality, Effectiveness and Safety, Houston, Texas, United States

## Abstract

Because nursing homes are both a residence and a treatment setting, care providers are faced with the challenge of balancing resident autonomy and safety on a daily basis. While there are standardized approaches for determining a capacity to make larger decisions such as providing consent for medical procedures, there are virtually no methods for assessing capacity to make everyday decisions (e.g., food choices, smoking, navigating outside the nursing home). While it is easier for staff to prevent residents from making decisions they deem risky, to truly offer person-centered care, it is important to support a resident’s right to make decisions if they have the capacity to do so. Currently, little is known about how nursing home staff conceptualize and determine everyday decision-making capacity and how that information is used in care planning. To understand the current processes and language nursing home staff use when considering a resident’s decision-making capacity, we conducted interviews with 37 staff at two Veterans Affairs (VA) Community Living Centers (VA-operated nursing homes; CLCs). Using qualitative content analysis, we coded the transcribed interviews and identified several overarching themes: autonomy vs. safety, communication (e.g., pathways, with caregivers, with residents), determining capacity (e.g., information gathering, assessment, assumptions, indicators, interdisciplinary team member roles, referrals), interventions (e.g., legal and staff-led), and terminology used. We will describe how the findings from this study can be used to tailor development and adaptation of tools to help nursing home staff assess resident everyday decision-making capacity and to incorporate the results into person-centered care approaches.

